# Five Cellular Genes as Candidates for Cervical Adenocarcinoma Molecular Markers

**DOI:** 10.3390/cancers17091558

**Published:** 2025-05-03

**Authors:** Isui Abril García-Montoya, Karla Berenice López-Córdova, Daniel Marrero-Rodríguez, Mauricio Salcedo-Vargas, Claudia Lucía Vargas-Requena, Angélica Maria Escárcega-Avila, Santos Adriana Martel-Estrada, Florinda Jiménez-Vega

**Affiliations:** 1 Instituto de Ciencias Biomédicas, Universidad Autónoma de Ciudad Juárez, Av. Plutarco Elías Calles #1210 Fovissste Chamizal, Ciudad Juárez 32310, Mexico; isui.garcia@uacj.mx (I.A.G.-M.); qarlalpz@gmail.com (K.B.L.-C.); cvargas@uacj.mx (C.L.V.-R.); maria.escarcega@uacj.mx (A.M.E.-A.); 2 Unidad de Investigación Médica en Enfermedades Endocrinas, Hospital de Especialidades Centro Médico Nacional Siglo XXI, Instituto Mexicano del Seguro Social, México City 06720, Mexico; dan.mar57@gmail.com; 3 Unidad de Investigación Biomédica y Oncológica Genómica, Hospital de Gineco Pediatría 3 A, OOAD CDMX Norte, Instituto Mexicano del Seguro Social, México City 07760, Mexico; masava89@gmail.com; 4 Departamento de Diseño, Instituto de Arquitectura, Diseño y Arte, Universidad Autonoma de Ciudad Juárez, Ciudad Juárez 32310, Mexico; adriana.martel@uacj.mx

**Keywords:** cervical adenocarcinoma, molecular markers, MACC1, RARβ, BCL2, HOXC8, E6/E7, genetic expression

## Abstract

Background/Aim: Cervical adenocarcinoma associated with Human Papillomavirus (HPV) infection represents 85–90% of all adenocarcinomas that have poor prognostic factors and is an important health public concern. Currently, cervical adenocarcinoma molecular markers are scarce. This study searched databases and the literature regarding candidate genes to find these molecular markers, which were experimentally evaluated in fresh cervical samples. Materials and Methods: Bioinformatic analysis of 161 transcriptomic libraries of cervical tissues with or without lesions from the NCBI database was performed using the Partek Genomics Suite 6.6v software. The selected genes with a *p* value of >0.05, and 1.5-fold change were considered. A search of molecular marker candidates of cervical lesions that were already published in the literature was performed. To validate the selected genes, total RNA from fresh cervical adenocarcinoma and cervical normal tissues were subjected to RT-PCR experiments; HPV detection was also performed. Results: Initially, twenty-five genes were identified using bioinformatic analysis, and their expression was evaluated. The results showed that the *HOXC6*, *HOXC8*, *RARβ*, *ELAVL2*, *URG4*, *CISD2*, *CA9*, *BCL2*, *Survivin*, *MACC1*, *CDKN2A*, and HPV *E6/E7* genes were found to be differentially expressed in CC. Among these, *RARβ*, *MACC1*, *BCL2*, *HOXC8*, and E6/E7/HPV exhibited higher statistical significance for CC samples. Conclusions: This five-gene panel could serve as a novel molecular tool for HPV-associated cervical adenocarcinoma detection.

## Simple Summary

This study describes the search for and evaluation of cervical adenocarcinoma molecular markers in a population of Ciudad Juárez, Chihuahua, México. Bioinformatic analysis of the NCBI database and 161 transcriptomic libraries was performed. The expression of selected genes was analyzed using semi-quantitative RT-PCR in samples from fresh cervical adenocarcinoma and cervical normal tissues. Five genes presented higher amplification frequency with a statistically significant difference, making them possible molecular markers for cervical cancer.

## 1. Introduction

Cervical cancer (CC) is one of the main cancer types for women, ranking fourth in incidence and mortality worldwide [1]. Currently, CC is diagnosed histopathologically using cervical smear and colposcopic procedures, but these approaches have the disadvantage of a long waiting period [2]. Moreover, several barriers are highly involved in early CC detection, such as culture, religion, health public services, etc., that make achieving an efficient program result more difficult [3]. Although the Papanicolaou test detects CC and, thus, helps in reducing its incidence, this test has relatively low accuracy and sensitivity [4]. Recent studies suggest that it is necessary to design novel systems or procedures for the actual CC screening programs.

The Federal Drug Administration (FDA) and the Pan American Health Organization (PAHO) [5] have already accepted the Human Papillomavirus (HPV) molecular test as an important test for CC screening. However, HPV is an essential factor but not sufficient for cervical carcinogenesis; a positive test result only indicates the presence of HPV sequences [6].

Squamous cell carcinomas represent 70% of CC cases [7], while the remainder are cervical adenocarcinomas. The prevalence of adenocarcinomas has been increasing over the years; it is currently the most recurrent in women above 30 years of age [8,9]. In addition, 80% of all adenocarcinoma cases are related to HPV types 16 and 18 [10,11].

Recently, the use of molecular markers associated with the distinctive characteristics of cancer has allowed for a more precise diagnosis, and therefore, it has helped to identify the best therapeutic approach to combat the diagnosed cancer type [12]. A molecular genetic marker is a sequence (gene, transcript, protein, metabolite) associated with a disease [13]. Some diagnostic or predictive panels have been developed for detecting different types of cancer [14,15,16]; however, panels for CC are scarce. Thus, the identification of genes differentially expressed in CC will facilitate the development of new diagnostic tools.

Moreover, is necessary to design novel molecular tools for future implementation with better predictive values and the ability to effectively identify women at risk of developing CC. This will reduce the burden of CC globally, especially in low-and middle-income countries [17].

The aim of this study was to identify candidate genes that identified the risk of cervical adenocarcinoma from the NCBI database and already-published papers and to evaluate their in vivo expression in fresh cervical adenocarcinomas.

## 2. Materials and Methods

### 2.1. Selection of Candidate Genes

To strengthen the present study’s methodology, bibliographic research and transcriptomic libraries were used to identify genes of interest. For this purpose, the inclusion criteria were as follows: genes that were identified in gene expression studies, genes that exhibited changes in expression throughout cervical carcinogenesis, reported for Cervical Intraepithelial Neoplasia grades 1–3 (CIN1–3) and CC, and genes that were differentially expressed in cancer cells. The exclusion criteria were studies carried out on cell lines and on treated CC patients. CIN2+ or high-grade and CIN3 Squamous Intraepithelial Lesions and in situ carcinomas are considered as high-risk cervical lesions.

For the bibliographic research, the PubMed database was used with the following keywords, cervical cancer, adenocarcinoma, molecular markers, and differential expression, and by applying the above-mentioned inclusion and exclusion criteria. After obtaining a list of related published papers that met the inclusion and exclusion criteria, the genes involved in cellular pathways related to the hallmarks of cancer [18] were selected and analyzed.

### 2.2. Identification of Candidates from Datasets

The transcriptomic library search for the cervical lesions was carried out with the Array Express database using the following criteria, including *Homo sapiens* transcriptomes, RNA assays, cervical tissues, and different stages of the carcinogenic process, as well as the Affymetrix Human matrix gene Chip U133. The obtained libraries were analyzed using the Gene Expression Omnibus (GEO), RRID:SCR_005012. After obtaining the transcriptomes, multiple comparisons were made using the Partek Genomics Suite 6.6v software, and the cutoff parameters were *p* = 0.05 and 1.5-fold change. Genes with the required level of statistical significance (*p* < 0.05) and a fold change cutoff of 1.5 were identified, and it was found that this combined criterion was significantly better for ranking candidate gene than *p*-value alone [19]. Fold change and statistical cut-offs modulate the outcome of microarray data, and these criteria suggest different biological meaning, with a fold change of 1.5 proving to be a better eliminator of background noise along with the *p*-value [20].

### 2.3. Biological Samples

Cervical samples were collected from women who attended the Colposcopy Clinic of the Sanitary Jurisdiction II at Ciudad Juárez, Chihuahua, Mexico. Women over 18 years old were invited to participate in the present protocol, all patients signed the informed consent letter, and a clinical history was obtained from each patient.

In total, ten CC samples biopsies and ten normal cervical scraping samples without lesions and free of HPV infection were used as a control. Patients without cervical lesions participated in the CC prevention program. The biopsies and scraping samples were reviewed by a pathologist immediately after collection, confirming the diagnosis. If more than 60% of epithelial cells were observed, then the samples were used for the detection of molecular gene expression.

### 2.4. DNA/RNA Extraction and cDNA Synthesis

DNA extraction from cervical samples was performed using the phenol/chloroform method and the extracted DNA was stored at −20 °C until use. Total RNA was extracted using TRIzol reagent and quantified using a Nanodrop 2000 device (Thermo Fisher Scientific, Wilmingtton, DE, USA). cDNA was synthesized with an ImProm-II Reverse Transcription System (Promega Co., Madison, WI, USA) as described by the manufacturer.

### 2.5. HPV Detection

**Figure 1:**
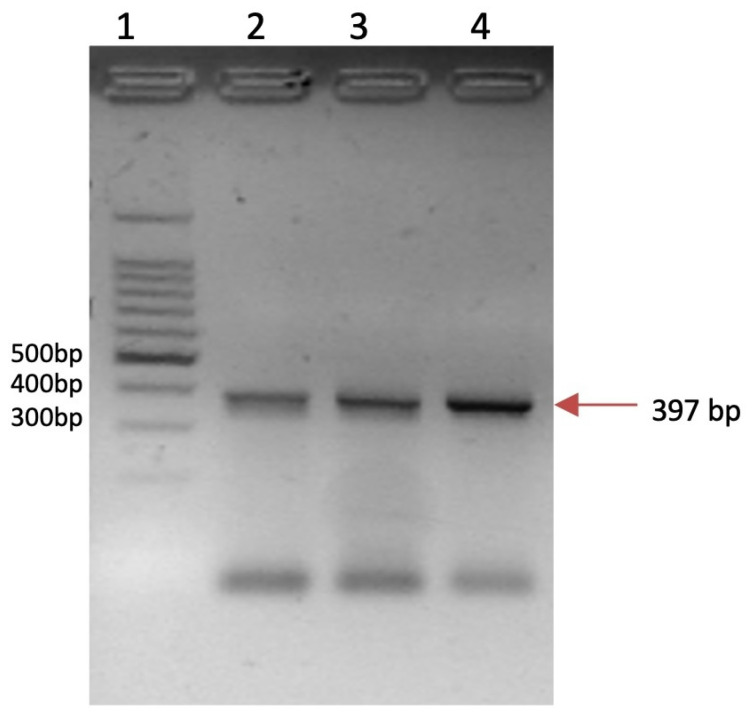
HPV 16 genotypification. Lane 1: molecular weight. Lane 2–4: positive samples.

The presence of the HPV in cervical samples was evaluated using the general gp5+/6+ primers directed to a region of the *hpv/l1* gene [21]. High-risk hpv16 sequences were identified by using specific primers [22] (Figure 1).

### 2.6. Evaluation of Gene Expression

The gene expression of selected genes was evaluated using semiquantitative PCR with specific primers for each gene. The obtained PCR products were detected using electrophoresis in an agarose gel with ethidium bromide staining. The gel images were analyzed using the EDAS 290 Kodak program (Eastman Kodak Company, Molecular Imaging Systems, Roachester, NY, USA) and densitometric analysis was performed to determine the net band intensity and, thus, the relative expression of each analyzed gene. The 18S rRNA constitutive gene expression was used to normalize the data. Table 1 lists the primer sets and amplification conditions.

### 2.7. Statistical Analysis

Based on the obtained results, the assumptions of normality and homogeneity of variance for each variable were analyzed using the Shapiro–Wilk and Levene statistical tests, respectively. Variables that did not meet the assumptions were transformed. The analysis of the relative expression data obtained was performed using Student’s *t* distribution test (with a significance level of 0.05), while Fisher’s exact test was performed to compare the proportion of amplification of each gene between the normal group and the cancer group (a significance level of 0.05).

## 3. Results

### 3.1. Overexpressed Gene Expression Identification from NCBI Analysis

The bioinformatics analysis of the NCBI database aided in selecting 21 genes from 26 published papers, as follows: *P63*, *URG4*, *HOXC6*, *HOXC8*, *RARβ*, *MCM7*, *PCNA*, *CISD2*, *IL-10*, *E6/E7*, *TAP73*, *COX2*, *CA9*, *MACC1*, *CTHRC1*, *BCL2*, *VEGF*, *CRABP1*, *cMYC*, *Survivin*, and *67LR* genes (Table 2). These genes were reported to be overexpressed in different types of cervical cancer at a statistically significant level, and they participated in several cellular pathways such as the cell cycle, cellular proliferation, immune system, apoptosis, angiogenesis, etc.

### 3.2. Identification of Overexpressed Gene from Transcriptomic Libraries for CIN2+

Using the Array Express platform data, 161 transcriptomic libraries were classified as CIN2+ accessed through the GSE63514 GEO repository [54] and GSE5787 [55]. Then, the data of the different cervical stages were compared with that of the normal group by using the Genomics Suite. After a stringent gene expression analysis (*p* ≤ 0.05), only the *CDKN2A*, *ZIC2*, *ELAVL2*, and *HS6ST2* genes were selected (Table 3). All these genes exhibited increased expression (>1.5-fold change) in the CIN2+ samples. Interestingly, these genes exhibited >2-fold expression for CIN2+ compared to CIN1, suggesting that they are potential and predictive cervical cancer markers.

### 3.3. Biological Samples and Characteristics

**Figure 2:**
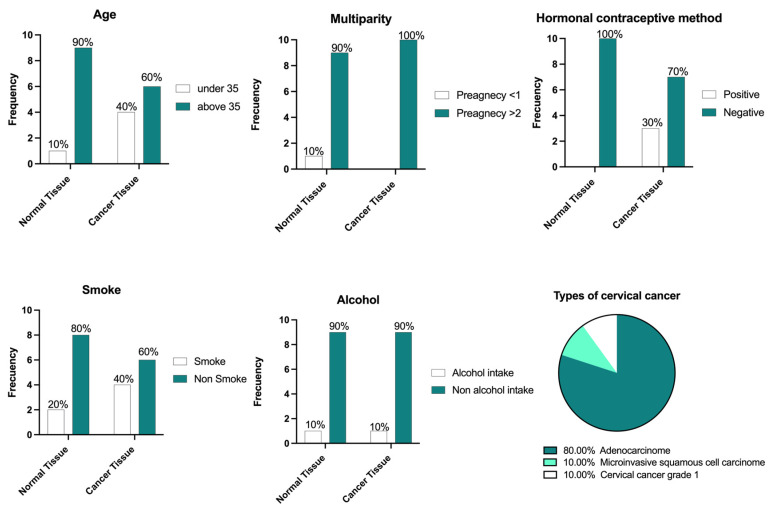
Clinical data of the patients.

The non-cancer patients were grouped according to their age, where 90% of these patients were over 35 years old, while cancer patients were mostly over 35 years old (60%); among these patients, 80% had histologically confirmed adenocarcinoma. Almost all the patients were multiparous with two or more pregnancies. Regarding the use of hormonal contraceptive methods, all patients with no lesions reported the use of hormonal treatments, but only 70% of women with cancer reported the use of hormonal treatments (Figure 2).

As for HPV infection, all cancerous samples were HPV16 positive, while the normal tissue samples were HPV negative.

### 3.4. Selection of Differentially Expressed Genes in Cervical Cancer

The 25 selected genes (21 from the literature and 4 from transcriptome databases) were grouped according to their role in cellular mechanisms: *URG4*, *P63*, *MCM7*, *PCNA*, *Tap73*, *CRABP1* *67LR*, *HS6ST2*, *ZIC2*, *HOxC6*, *HOXC8*, *RARB*, *E6/E7*, *CDKN2A*, and *ELAVL2* for cell cycle, cell division and proliferation; *Survivin*, *BCL2*, and *CISD2* for apoptosis; *COX2*, *CTHRC1*, *VEGF*, and *cMYC* genes for angiogenesis; *MACC1* and *CA9* for invasion and metastasis; and *IL-10* for anti-inflammatory response.

**Figure 3:**
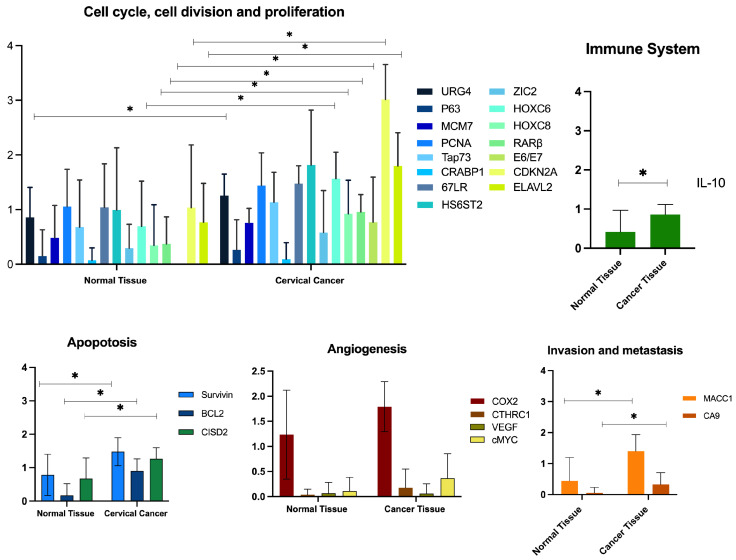
Relative expression of evaluated genes in normal and cancerous tissues, classified by hallmarks of cancer. * Significant differences.

To validate the selected genes, total RNA was subjected to RT-PCR assays in normal and CC samples. As can be observed in Figure 3, even when the bioinformatics analysis indicated differential expression, intriguing in vivo evaluation results were observed. Finally, only 13 genes were in vivo differentially expressed (Table 4): *HOXC6*, *HOXC8*, *RARβ*, *E6/E7*, *CDKN2A*, *ELAVL2*, *URG4*, *CISD2*, *CA9*, *BCL2*, *Survivin*, *MACC1*, and *IL-10*.

**Figure 4:**
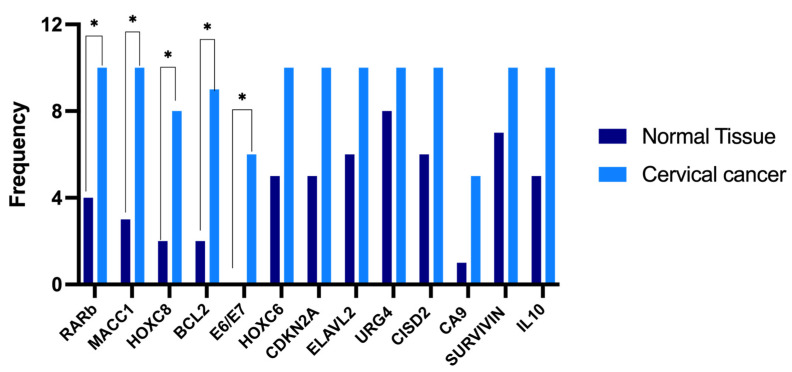
Frequency of expression of genes in normal and cancer samples. * Statistical difference.

The frequency of expression of the 13 genes was evaluated in normal and cancer samples, and it was found that only 5 genes were expressed more often in CC. These genes are *MACC1*, *HOXC8*, *BCL2*, *RARβ*, and the oncoproteins *E6/E7*. Figure 4 shows the expression frequency of the 13 genes among normal and cancer samples.

## 4. Discussion

Thirteen genes were found to be differentially expressed in cervical adenocarcinoma samples using microarray databases and literature reports, but only the *MACC1*, *HOXC8*, *BCL2*, and *RARβ* genes were the most representative of the expressed genes. These candidate genes could be considered for detecting cervical adenocarcinomas, as they are involved in the cellular division and proliferation, invasion, apoptosis, and immune system hallmarks.

Recently, the Global Strategy for CC Elimination Initiative was announced by the WHO. This initiative endeavors to screen 70% of women globally using a high-performance test [56]. However, the success of this initiative will depend on access to public health services in each region.

The role of HPV in cancer is widely known [57]. It is accepted that the molecular mechanism of cervical epithelial transformation entails E6/E7 viral oncoprotein expression, where p53 and Rb suppressor proteins are targeted by these viral oncoproteins. As expected, *E6/E7/HPV* gene expression was over-represented in the cervical carcinoma samples. This is supported by studies indicating that *E6/E7* RNA expression is a valuable molecular marker tool to identify CIN2+ detection or women at high risk of developing cervical cancer [58,59]. Furthermore, it has been reported that HPV-16 infection is associated with cervical adenocarcinoma [60]. Currently, commercial tests such as PreTect HPV-Proofer 7^®^ or the macro/micro test are already available for assessing viral expression [61,62]. As *MACC1*, *HOXC8*, *BCL2*, and *RARβ* are overexpressed genes, we hypothesize that these genes are related to or indirectly influenced by viral oncoproteins. Moreover, it has been previously reported that HPV oncoproteins enhance *RARβ* expression [63].

Basic research on the transformation of cervical keratinocytes has aided in comprehending the expression of the HOX homeotic gene family, including the *HOXC8* and *HOXC5* genes [64,65]. In this case, genes modulated in adenocarcinomas, such as HOXC genes, could be directly related to and activated by viral sequences [65]. Thus, the evidence on the homeotic gene’s role in cancer shows that the cellular differentiation epithelial mechanisms are directly related to cervical adenocarcinomas via HPV infection [66]. Furthermore, there is evidence that HPV-16 infection modulates the HOXC genes via the E7 oncoprotein, with H3K4me*3* and H3K27me3 as the gene promoters [67].

One of the most important transcription factors studied in cancer is the *cMYC* gene, and its important role in cell proliferation has been demonstrated [68]. Even though no statistical significance was observed, its role in cancer is important [69].

Recently, it has been proposed that *MCM7* gene expression could be a prognostic factor in breast luminal cancer [70]. The *MCM7* gene could play an important role in cervical cancer cells, allowing cellular replication. For its promoter activity, the *E2F* transcription factor is necessary. In HPV-infected cells, the HPV/E7 protein releases the *E2F* transcription factor from the Rb-E2F complex, promoting cell growth and cellular function [71].

*BCL2* expression is involved in apoptosis, and its overexpression inhibits apoptosis [72,73]. Furthermore, it has been proven that *BCL2* expression is a predictor of neoadjuvant chemotherapy in urothelial bladder and breast cancer [74,75]. According to this study, *BCL2* expression could be useful as a marker and a predictor of neoadjuvant chemotherapy for adenocarcinomas [76].

Regarding the contrasting results, where not all of the selected genes were overexpressed in cancer samples, they can be explained in part by the fact that the obtained statistically significant *p*-values were not necessarily representative. A limitation of this study is the small number of fresh samples used to validate the candidate genes. We hypothesize that, even with the small number of samples used (randomized selection), there is strong evidence that the selected genes, *HOXC6*, *HOXC8*, *RARβ*, *BCL2*, and *E6/E7*, can be used as a pan early cervical adenocarcinoma test. The lack of correlation in the gene overexpression of CC samples could be explained by the intra/inter-heterogeneity of the samples.

There is evidence that *MACC1* overexpression predicts a poor clinical outcome of hepatitis B virus-related hepatocellular carcinoma [77]. This could suggest that *MACC1* expression is a viral target. Evidently, TNF-α regulates the induction of *MACC1* via NF-κB and the transcription factor c-Jun in an inflammatory environment [78]. In cervical cells harboring the HPV sequences in an inflammatory environment, the MACC1 gene expression could be involved in virus infections.

Finally, there is enough information on the role of *CDKN2A*, *ZIC2*, *ELAVL2*, and *HS6ST2* genes in cancer. Thus, they could be considered as important molecular markers useful for cervical screening programs in CIN*2+* high-risk cervical samples. A major limitation of this study was the small number of samples used; therefore, it is necessary to conduct studies with a larger number of samples to validate the study data. Efforts are being taken to identify distinctive molecular makers that help in the early diagnosis of diverse types of cancer, and the results of these efforts could depend on the variability of cancers, populations, and risk factors. Thus, it is necessary to continue research to find specific molecular markers that help reduce the incidence rate of cancer.

## 5. Conclusions

In conclusion, this exploratory pilot study, through its robust and holistic analysis, provides evidence that *MACC1*, *HOXC8*, *RARβ*, *BCL2*, and *E6/E7* could be promising molecular markers for the detection of cervical adenocarcinomas. In addition, the *CDKN2A*, *ZIC2*, *ELAVL2*, and *HS6ST2* genes can be used in the screening of CIN*2+* cervical samples.

## Figures and Tables

**Table 1:** Details of nucleotide primers used in this study.

Gene Symbol	Primer Sequence (5′-3′)	Tm °C	Amplicon Length (pb)	Identification Number/Reference
*URG4*	Fw GCATCAGAGAGACGAACAGC	62	180	NM_017920.4
	Rv GCACGTCCAGCACCATAG			
*P63*	Fw GAGCTGAGCCGTGAATTC	55	319	AB082923.1
	Rv CCTTCCTGTCTCTTCCTGG			
*HOXC6*	Fw GAGGAAAAGCGGGAAGAG	60	250	NM_004503.4
	Rv CGTGGTGAAAGAGAGTTGTG			
*RARβ*	Fw GTGTCCTTCCTGATTCATGC	62	163	[23]
	Rv CCACTCTACCACAGCTTTCAC			
*MCM7*	Fw GCTGCATTGATGAGTTCG	45	271	NM_005916.4
	Rv CGTAGGTCATTGTCTCGG			
*PCNA*	Fw CTCCCAAGATCGAGGATG	55	249	NM_002592.2
	Rv GACCAGATCTGACTTTGGAC			
*CISD2*	Fw GGCTGCTGCAATTTGAAG	57	264	NM_001008388.5
	Rv GTGTACGGAGGGTCAACTG			
*IL-10*	Fw CCATTCCAAGCCTGACCAC	60	181	[24]
	Rv GAATCCCTCCGAGACACTG			
*E6/E7*	Fw ACCGAAAACGGTTGAACCGAAAACGGT	60	500	[25]
	Rv GAG CTG TCG CTT AAT TGC TC			
*TAP73*	Fw GAGCAGTACCGCATGACC	65	290	NM_005427.4
	Rv CGTGAACTCCTCCTTGATG			
*COX2*	Fw GCTGTATCCTGCCCTTCTG	55	291	AY462100.1
	Rv CGGGAAGAACTTGCATTG			
*CA9*	Fw CGGCTACAGCTGAACTTCC	60	238	NM_001216.3
	Rv GTAGCTCACACCCCCTTTG			
*MACC1*	Fw CAATGGAAGCCCTTTTGC	60	247	NM_182762.4
	Rv GGTGACGGAAGAGCTTTAGC			
*HOXC8*	Fw GAGCTCCTACTTCGTCAACC	55	250	NM_022658.4
	Rv GTCTCCGTGGCAGCTAAG			
*CTHRC1*	Fw GGACACCCAACTACAAGCAG	55	380	NM_138455.4
	Rv CCAGCACCAATTCCTTCAC			
*BCL2*	Fw CGACTCCTGATTCATTGGG	55	550	NM_000633.2
	Rv GCTTTGCATTCTTGGACG			
*VEGF*	Fw CTTCAAGCCATCCTGTGTGC	55	147	[24]
	Rv GCTCATCTCTCCTATGTGC			
*CRABP1*	Fw GCACGCAAACTCTTCTTGAAG	60	133	[26]
	Rv CGGACATAAATTCTGGTGCAG			
*cMYC*	Fw CCTCAACGTTAGCTTCACC	65	242	NM_002467.6
	Rv GAAGGGAGAAGGGTGTGAC			
*SURVIVIN*	Fw GTCCCTGGCTCCTCTACTG	65	222	NM_001168.3
	Rv CACTGGGCCTGTCTAATCAC			
*67LR*	Fw GGCTGTGCTGAAGTTTGC	57	216	NM_002295.6
	Rv CCACATAGCGCAGAGGAG			
*CDKN2A*	Fw GAAGGTCCTACAGGGCCACA	68	211	NM_000077.4
	Rv CAACACAGTGAAAAGGCAGAAGC			
*ELAVL2*	Fw GACAAACTATGATGAGGCTGC	68.1	330	NM_004432.5
	Rv CCCTGTCCTCTTGTCCATATTC			
*HS6ST2*	Fw CGTACCGCTCGGAGGATG	63.5	313	NM_001077188.2
	Rv GTGAGCTCGGTCCAGTCG			
*ZIC2*	Fw GGAGCAGAGCAACCACGTC	64.5	268	NM_007129.5
	Rv GTGCATGTGCTTCTTCCTGTC			
*18S*	Fw TTTGCGAGTACTCAACACCA	60	280	[27]
	Rv GTTGTCCSGSCCSTTGGCTA			

**Table 2:** Differentially expressed genes in cervical cancer reported in the literature.

Protein	Gene Name	Molecular Function/Biological Process	Type of Cancer	*p*-Value	Reference
Tumor protein p63	*p63*	DNA binding/transcription, transcription regulation	Cervical cancer	0.001	[28]
Minichromosome maintenance complex component 7	*MCM7*	DNA binding/cell cycle	Cervical cancer, CIN 3, invasive cancer	0.002, 0.035	[29,30,31]
Upregulator of cell proliferation	*URG4*	Proliferation	Cervical cancer	0.0001	[32]
Retinoic acid receptor beta	*RARβ*	DNA binding/transcription, transcription regulation	Cervical cancer	NR	[33]
Vascular endothelial growth factor C	*VEGFC*	Growth factor/angiogenesis	Cervical cancer	0.002	[34]
Interleukine 10	*IL-10*	Cytokine	Invasive squamous cell carcinoma of the cervix	<0.05	[35]
BCL2 apoptosis regulator	*BCL-2*	Apoptosis	Cervical cancer	<0.001	[36]
CDGSH Iron-Sulfur Domain-Containing Protein 2	*CISD2*	RNA binding/Autophagy	Cervical cancer	<0.001	[37]
Cyclooxygenase 2	COX-2	Angiogenesis	Cervical cancer	0.0152	[38]
Tumor suppression protein P73	TAP73	P53binding/positive regulation apoptosis process	Cervical cancer	0.001	[39]
Carbonic anhydrase 9	CA9	Proliferation	Uterine cervical cancer	0.008	[40]
Survivin	SURVIVIN	Apoptosis	Cervical cancer, squamous cell carcinomas	0.0001 <0.05	[41,42]
Laminin Receptor 67 kD, Ribosomal Protein SA	*67LR*	Laminin binding/cell adhesion	Squamous cell carcinomas, carcinoma in situ	0.0001	[43]
Myc proto-oncogene protein	*cMYC*	DNA binding transcription factor/proliferation	Cervical cancer, squamous cell carcinoma	<0.0001 <0.05	[44,45,46]
Collagen triple helix repeat-containing	*CTHRC1*	Cell migration	Squamous cell carcinoma	<0.001	[47]
Proliferating cell nuclear antigen	*PCNA*	DNA binding/DNA replication	Squamous cell carcinoma	NR	[48]
MET Transcriptional Regulator MACC1	*MACC1*	Growth factor activity/transcription regulator	Cervical cancer	0.039	[49]
Homeobox protein Hox-C6	*HOXC6*	DNA binding/transcription regulator	Cervical cancer	0.016	[50,51]
Homeobox protein Hox-C8	*HOXC8*	DNA binding/transcription regulator	Cervical cancer	<0.0001	[52]
Cellular retinoic acid-binding protein 1	*CRABP 1*	Cell cycle	Cervical cancer	<0.001	[53]
Proteins E6/E7	*E6/E7*	DNA binding/transcription regulation, modulation of host cell apoptosis	Cervical cancer	0.034	[25]

**Table 3:** Differential expression according to the stage of cervical intraepithelial neoplasia.

	Classification			
Gene	CIN I	CIN II	CIN III	Cancer
*CDKN2A*	2.91	7.99	11.11	12.49
*ZIC2*	1.38	2.12	4.05	13.15
*ELAVL2*	2.11	2.92	4.37	7.29
*HS6ST2*	2.67	2.90	6.28	6.51

Expression level does not have explicit unit.

**Table 4:** *p*-values of evaluated genes of hallmarks of cancer.

Hallmark of Cancer	Gene	*p*-Value
Cell cycle, cell division, and proliferation	*URG4*	0.0395 *
	*P63*	0.3191
	*MCM7*	0.1041
	*PCNA*	0.0974
	*Tap73*	0.0889
	*CRABP1*	0.4246
	*67LR*	0.0680
	*HS6ST2*	0.0511
	*ZIC2*	0.1618
	*HOXC6*	0.0060 *
	*HOXC8*	0.0373 *
	*RARβ*	0.0031 *
	*E6/E7*	0.0078 *
	*CDKN2A*	0.0001 *
	*ELAVL2*	0.0013 *
Immune system	*IL-10*	0.0190 *
Apoptosis	*Survivin*	0.0047 *
	*BCL2*	0.0001 *
	*CISD2*	0.0086 *
Angiogenesis	*COX2*	0.0524
	*CTHRC1*	0.3900
	*VEGF*	0.4728
	*cMYC*	0.0859
Invasion and metastasis	*MACC1*	0.0024 *
	*CA9*	0.0326 *

* Statistical difference.

## Data Availability

The authors confirm that the data supporting the findings of this study are available within the article.
